# Evaluation and optimization of ecological spatial resilience of Yanhe River Basin based on complex network theory

**DOI:** 10.1038/s41598-024-51966-z

**Published:** 2024-01-16

**Authors:** Quanhua Hou, Qingze Li, Yuxuan Yang, Jizhe Zhou, Yang Du, Yahui Zhang

**Affiliations:** 1 School of Architecture, Chang’an University, Xi’an, 710061 China; 2 Engineering Research Center of Collaborative Planning of Low-Carbon Urban Space and Transportation, Universities of Shaanxi Province, Xi’an, 710061 China; 3 West Airport Group, Xi’an, 710049 China; 4 School of Human Settlements and Civil Engineering, Xi’an Jiaotong University, Xi’an, 710049 China

**Keywords:** Ecology, Environmental sciences

## Abstract

The loess hilly and gully areas have broken terrain, vertical and horizontal ravines and fragile ecological environments. Improving the resilience of the regional ecological space is conducive to improving the quality of the local ecological environment. With the ecological space of the Yanhe River Basin selected as the research object, this paper constructs a research framework of "network identification topology-resilience evaluation-spatial optimization" and uses morphological spatial pattern analysis (MSPA) and the minimum cumulative resistance model (MCR) to identify ecological spatial networks. Based on circuit theory, the ecological pinch point is identified, the ecological spatial network is optimized, and scenario simulation is performed. Through complex network theory and related indicators, the ecological spatial resilience of the basin is evaluated, and the hierarchical optimization strategy of the ecological space is confirmed. According to the ecological function of the source area and the results of the resilience evaluation, the boundaries of the protected control area, guidance development area, remediation area, and maintenance and improvement area of the basin are delineated. The importance of ecological source and corridor protection is classified, and corresponding protection strategies are proposed. The research results can provide theoretical support and practical guidance for the territorial spatial planning and ecological space construction of the Yanhe River Basin and provide a reference for the ecological restoration, resource development and environmental governance of the Yanhe River Basin.

## Introduction

The Yanhe River is a first-class tributary of the Yellow River, flowing into the Yellow River from the northwest to southeast. As a typical loess hilly and gully region, the soil erosion problem in the Yanhe River Basin is particularly prominent. The loess hilly and gully region is broken, and it has horizontal ravines. The fragile ecological environment in the region, coupled with the strong human activity interference, makes it one of the regions with the most serious soil water erosion in China and even in the world^1^. Since the large-scale implementation of the Grain for Green (GFG) program in 1999^2^, regional vegetation coverage has greatly improved^3^. However, the ecological environment has not significantly improved. There are two main reasons. First, large-scale urban construction and environmental governance have caused the fragmentation of the ecological patch area and have ruptured the ecological corridor in the region^4^. Second, Excessive ecological construction in non-key areas consumes limited water resources in the region, resulting in unstable vegetation restoration and deterioration of environmental quality. Restoring local ecological environment is supposed to be a sustainable and continuous process, during which a resilient mindset of continuous adaptation and active response is required. Cumming et al. proposed the concept of spatial resilience^5,6^, and the combination of resilience and spatial optimization can better find ways to optimize the quality of ecological environment. They integrated resilience theory into the framework of landscape ecology and used resilience indicators to quantify spatial attributes such as connectivity and the importance of spatial location. Connectivity conservation is critical for managing healthy ecosystems, preserving biodiversity, and adapting to climate change across all biological communities and spatial scales^7^. The maintenance and improvement of key nodes are also important factors influencing the impact of ecological spatial resilience^8^. Therefore, it is urgent to evaluate and optimize the resilience of the ecological space in the region. Basin is the basic spatial unit of natural landforms and soil and water conservation management on the Loess Plateau^9^. As a sensitive and complex independent ecosystem^10^, it provides ecosystem services such as water conservation and biodiversity maintenance. Improving the quality of the ecological environment of a basin involves maintaining stable ecological space and the ability to adapt to risks when human interference occurs. In the face of complex regional characteristics and rapid changes, related studies have shortcomings in terms of assessing the complexity and dynamics of the indicators. Therefore, the complex network method and index are used to solve this problem. Relevant studies lack a better solution for the identification of important regions in complex systems.

At present, resilience evaluations usually need to convert system information into indicators and then use the indicators to measure the level of attributes, functions or capabilities^11^. The most typical model is the rapid evaluation model proposed by Kristine. In addition, evaluation methods for ecological spatial resilience include the comprehensive index method that is focused on surface conditions and atmospheric pollution^12^, the entropy weight technique for order preference by similarity to ideal solution (TOPSIS) to assign a weight to each evaluation index^13^, the pressure-state-response (PSR) method to reveal links between systems and human activity^14^, and the substitution index method to characterize physical conditions, human activities and ecological security patterns (ESP)^15^. Zhang et al. used the comprehensive index method to evaluate different stages of ecological resilience in the red soil area in southern China^12^. Tang et al. used TOPSIS to measure the ecological resilience of 117 resource-based cities over nine years^13^. According to Xie et al., the PSR model was used to evaluate the level of rural ecological resilience in Weiyuan County in 2021^14^. However, these three methods cannot reflect the connectivity of ecological space. Yuan et al. used the substitution index method to study the current resilience distribution in Changzhi. The evaluation results of grid units and ESP units were superimposed and analysed to solve the imbalance between mineral resource development and ecological protection^15^. However, evaluations of indicators of complex changes in ecological space are lacking. The goal of ecological land space is to improve ecological functions and restore damaged ecosystems. Thus, basin ecological spatial resilience includes ecological background resilience and ecological process resilience. Janssen et al. showed that ecosystems have spatial network characteristics^16^. The plaque and corridor elements contained in ecological space correspond to the node and edge elements in the network. The ecological patches represent ecological land with good ecological environment quality, and the corridors are the channels formed in the processes of species migration and energy flow. Thus, network resilience can reveal spatial resilience. On the one hand, network resilience is manifested as the ability of the network structure itself to resist shocks, that is, static resilience. On the other hand, network resilience is expressed as the ability of the network to resume normal operation after being disturbed, that is, dynamic resilience. Thus, static resilience can embody diversity, collaboration, interdependence, stability and connectivity principles, and dynamic resilience can embody redundancy and adaptability principles^17^. Nevertheless, current research lacks methods for identifying key elements in the network. Moreover, the current ecological spatial network-related research focuses on network structure identification and ecological spatial planning, ignoring the simulation prediction of possible future scenarios.

The complex network method can highly abstract real and complex systems and identify key modules in a system. It is a powerful tool for studying complex sciences and systems^18^. A variety of topological indicators can be used to characterize ecological spatial networks^19^. Zhang et al. used node degree, betweenness, and clustering coefficient characteristic indicators to evaluate grid structure performance^20^. Zhou et al. adopted node degree, structural hole, clustering coefficient, k-core and core–edge, and betweenness feature metrics to assess small watershed ecological spatial network resilience^10^. Zhang et al. used a connection robustness and restore robustness assessment to study the dynamic characteristics of network efficiency and connectivity changes under malicious and random attacks^21^. Therefore, complex network theory has a rich index system for network evaluation. Empirical research on the evaluation and optimization of ecological spatial resilience based on complex networks for basins with prominent human-land conflicts needs to be carried out.

## Results

### Identification of the ecological spatial network

**Figure 1:**
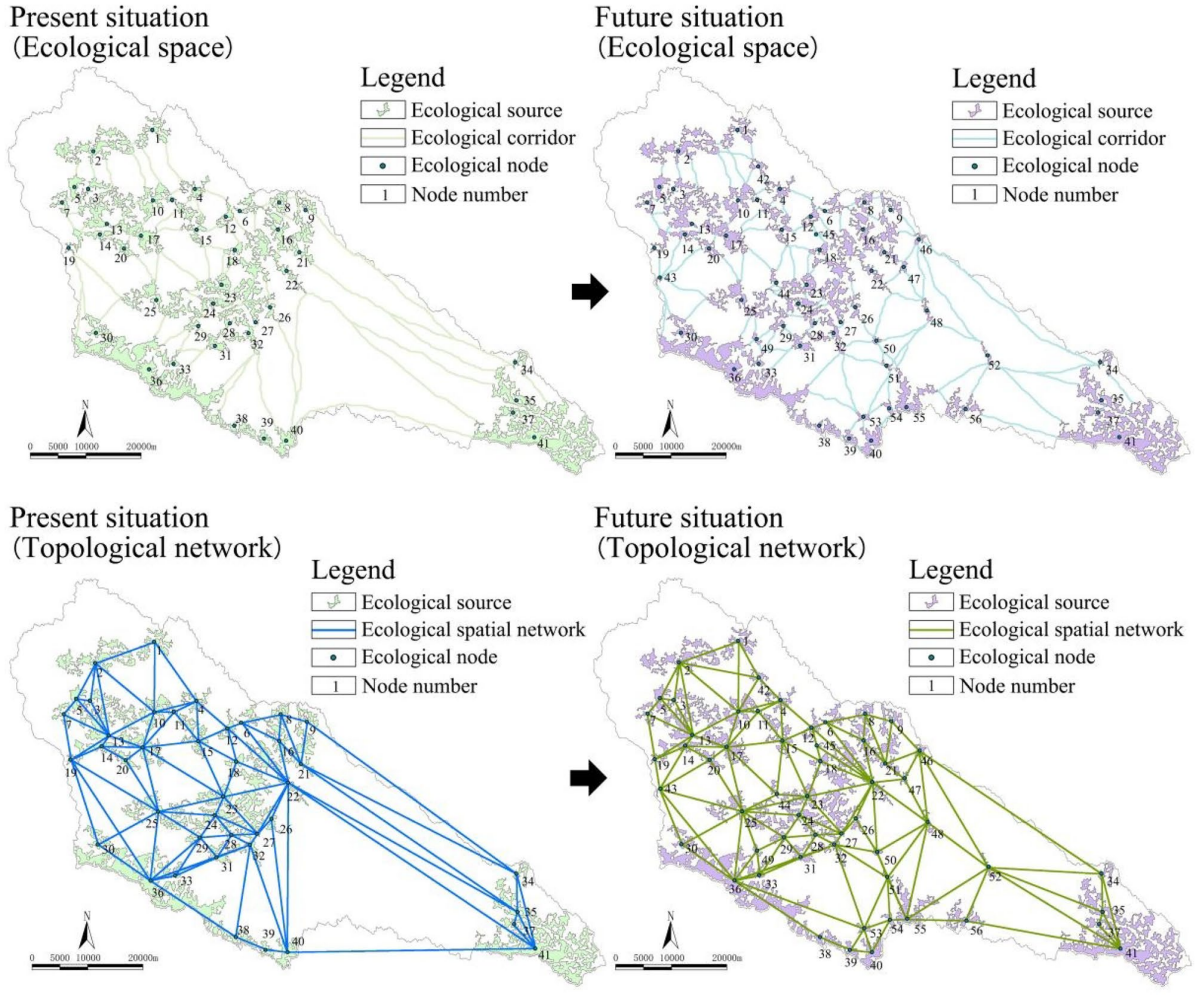
Ecological space and network topology.

The recognition results of the ecological spatial network in the Yanhe River Basin are shown in Fig. 1. Currently, the watershed has 41 ecological source regions. A total of 75.61% is distributed in a planar shape in areas with good water conservation, such as central and western reservoirs, ecological forests, and economic forests, and there is high connectivity between source areas. A total of 14.63% is distributed in a band shape on the southern edge of the mountain, and the rest of the sources are in the eastern edge of the watershed. The properties of each node are shown in Table 1. The watershed has 82 ecological corridors, which are distributed along the water systems, valleys, forest belts, and mountains on both sides of the road. Fifteen ecological nodes were added to the scenario simulation of the scheme. The attributes of the new nodes are shown in Table 2. Fifty-nine ecological corridors were added to achieve more effective node organization and connection and to improve the ecological connectivity of the watershed. Among them, nodes 42, 43, 47, 49, 53, 54, and 55 were formed based on the natural development scenario, nodes 46, 48, 51, and 52 were formed based on the ecological priority scenario, and nodes 44, 45, 50, and 56 were formed based on the economic priority scenario.

**Table 1:** Node list of the original ecological source areas in the Yanhe River Basin.

Characteristic	Node number
Ecological forest	1, 2, 5, 6, 7, 8, 9, 10, 12, 13, 14, 16, 18, 19, 21, 22, 24, 25, 26, 27, 28, 29, 30, 32, 34, 35, 36, 37, 38, 39, 40
Heiquanyi grottoes	3
Jianxia temple grottoes	4
Qianfo temple grottoes	11
Majiagou reservoir	15
Wangyao reservoir	17
Ecological forest, Economic forest	20
Fangjiahe reservoir	23
Wanhua mountain scenic spot	31
Fodaoping scenic spot	33
Angou reservoir	41

**Table 2:** Node list of new ecological source areas in the Yanhe River Basin.

Node number	Characteristic	Development scenario
42	Ecological forest	Natural development scenario
43	Ecological forest	Natural development scenario
44	Economic forest	Economic priority scenario
45	Economic forest	Economic priority scenario
46	Suntai Reservoir	Ecological priority scenario
47	Ecological forest	Natural development scenario
48	Ecological forest	Ecological priority scenario
49	Ecological forest	Natural development scenario
50	Economic forest	Economic priority scenario
51	Ecological forest	Ecological priority scenario
52	Wazhuang Reservoir	Ecological priority scenario
53	Ecological forest	Natural development scenario
54	Ecological forest	Natural development scenario
55	Ecological forest	Natural development scenario
56	Qili Village Grottoes	Economic priority scenario

### Resilience evaluation of the ecological spatial network

#### Static resilience evaluation

##### Diversity

**Figure 2:**
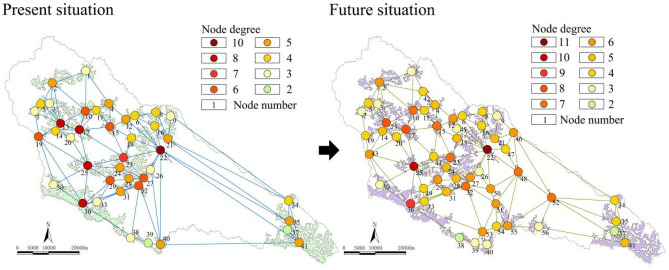
Node degrees and changes in the complex network.

As shown in Fig. 2, the average degree value of the ecological node of the Yanhe River Basin was 4.83. There were no isolated nodes with a node degree of 0 in the network. The maximum node degree was 10. The node degree of 46.34% of nodes was less than or equal to 4. The ecological nodes starting from the ecological forest (13) and passing through Wangyao Reservoir (17), economic forest (25), ecological forest (36), Fangjiahe Reservoir (23) and economic forest (22) had a high node degree and were connected in pieces, and they can provide good ecological service functions. However, ecological nodes in the eastern part of the basin were less connected with other nodes. The average node degree value of the optimized ecological space network was 5.04, which increased by 0.21 compared with the status quo. Moreover, diversity increased by 4.34%. The highest node degree of the optimized network was 11, accounting for 1.78%. Forty-eight percent of the nodes had a degree less than or equal to 4. In contrast, the highest node degree value increased, and the network was more uniform. The protection and construction of Wazhuang Reservoir (52) and ecological forest (49, 51, 53, 55) land can significantly enhance the connectivity of ecological space.

##### Collaboration

**Figure 3:**
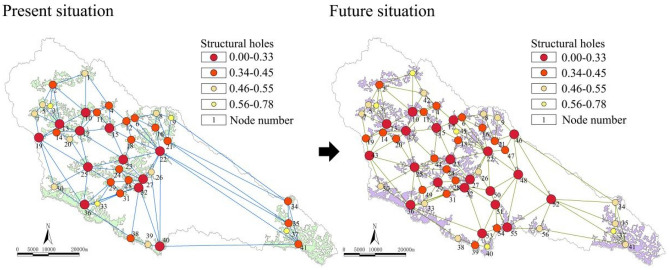
Structural holes and changes in the complex network.

As shown in Fig. 3, the structural hole of the current basin is mainly located in the ecological space at the edge of the basin, which is usually located on the slope at the edge of a watershed. The structural hole values of the Qianfo Temple Grottoes (10), Majiagou Reservoir (15), Wangyao Reservoir (17), Fangjiahe Reservoir (23), economic forest (25), and ecological forest (13, 19, 22, 27, 32, 36, 40) nodes in the basin were low, which helped to form a more effective “network” connection between ecological source regions in the area. Therefore, attention should be given to the protection and development of these nodes. With 0.56 as the threshold, the proportion of nodes located in the structural hole to the structural hole in the area was 9.76%. After optimization, the structural hole ratio of the ecological space network was reduced to 8.93%, and the collaboration was increased by 0.83%, thus forming a more effective ecological connection.

##### Interdependence

**Figure 4:**
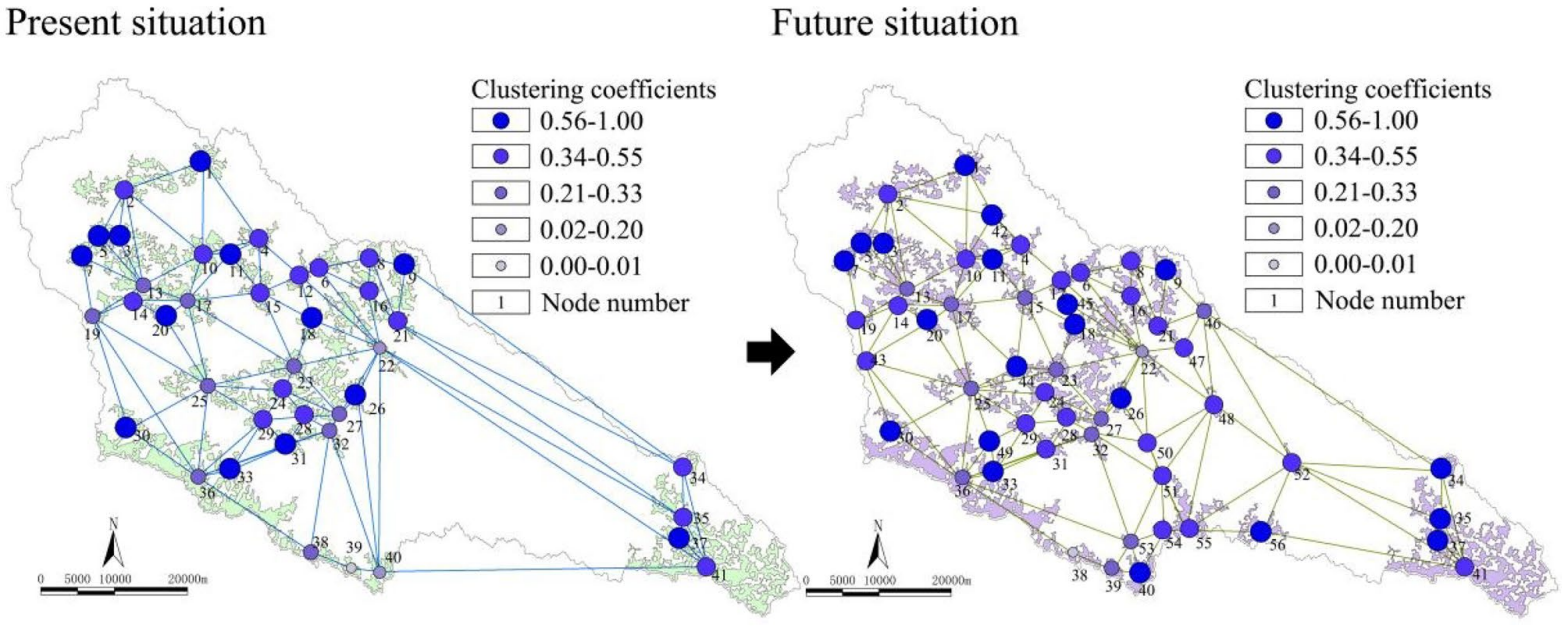
Clustering coefficients and changes in the complex network.

As shown in Fig. 4, in the future, the ecological space of the basin will form four clusters of nodes around the Wangyao Reservoir (17), ecological forest (22) and the Fangjiahe Reservoir (23), ecological forest (36), and the Angou Reservoir (41). After optimization, the proportion of nodes with lower clustering coefficients was reduced to 23.21%, and the interdependence performance was improved by 6.06%. Optimizing the primary ecological node is one of the important ways to improve interdependence.

##### Stability

**Figure 5:**
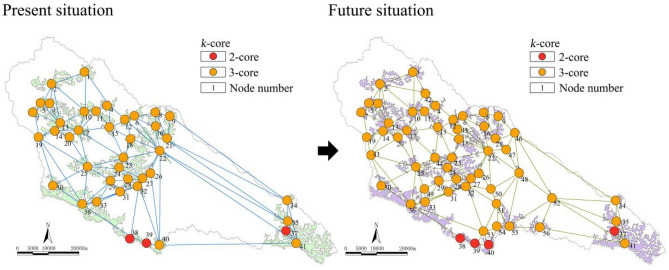
k-core and changes in the complex network.

**Figure 6:**
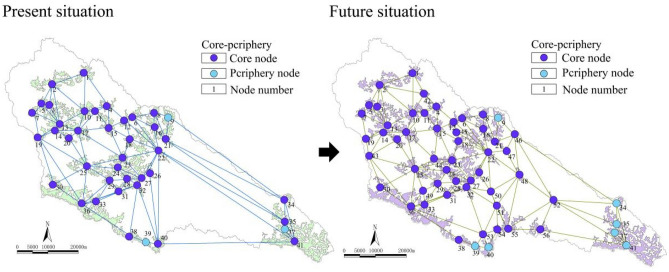
Core-periphery and changes in the complex network.

As shown in Figs. 5 and 6, the maximum value of the complex network “k-core” in the Yanhe River Basin was 3-core. The ecological stability of the 3-core area was higher. Most of the watersheds were 3-core nodes. Only the Angou Reservoir (41) and ecological forest (38, 39) nodes in the south and east of the basin were 2-core. The 2-core network vulnerable areas accounted for 7.32%. At present, the number of core nodes in the network is 38, and they are ecological forest and economic forest. In the future, the 3-core area will expand. The proportion of regional ecological nodes reached 92.86%. There were only four 2-core nodes in the south and southeast of the basin, with a ratio of 7.14%. Furthermore, the stability improved by 0.18%. There were a total of 49 core nodes in the network. With the further expansion of the core area in the watershed, new core areas appeared in the middle reaches and in the south of the watershed, which enhanced the energy flow between substances and increased the stability of the ecological space.

##### Connectivity

**Figure 7:**
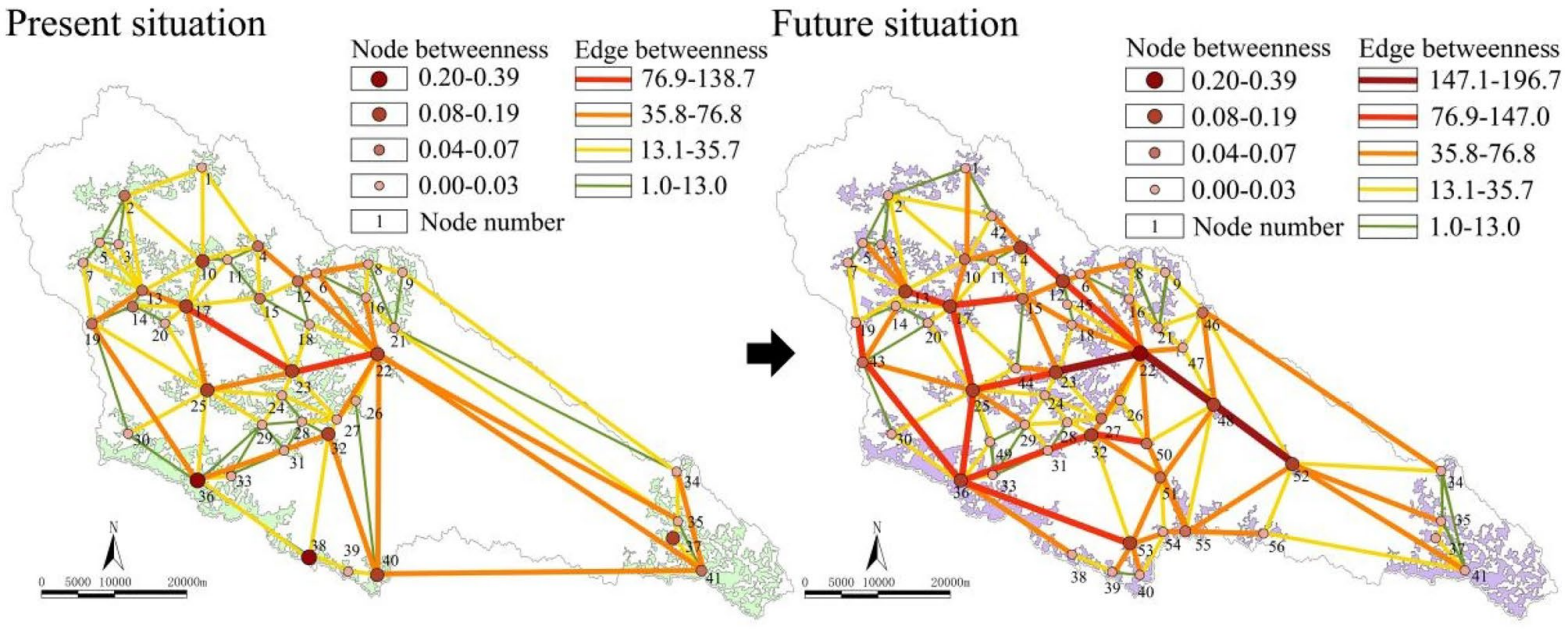
Nodes/edge betweenness and changes in the complex network.

As shown in Fig. 7, the main ecological corridors of the basin were divided into two lines from west to east. Both corridors departed from the Angou Reservoir (41). The ecological corridors in the middle passed through the ecological forest (22), Fangjiahe Reservoir (23), Wangyao Reservoir (17) and ecological forest (13) and finally reached ecological forest (19). The ecological corridor on the south side started from the Angou Reservoir (41), passed through the ecological forest (32, 40), Wanhua Mountain Scenic Area (31) and ecological forest (36), and finally merged with the central corridor in the ecological forest (19). In the future, the role of the ecological corridor along the river will gradually increase. A new hub corridor will be generated, which will depart from the Wazhuang Reservoir (52), pass through ecological forest (22) and new ecological forest (48) and arrive at the Fangjiahe Reservoir(23). In addition, a mountain landscape corridor connected with the ecological forest (43, 36, 53) will appear. The overall connectivity computed based on the betweenness value improved by 16.37%. Ecological nodes 4 and 6 located in the forest area on the north side and ecological nodes 13, 15, 17 and 25 located in the middle will be promoted as hubs, thereby enhancing the overall connectivity of the network.

#### Dynamic resilience evaluation

##### Redundancy

**Figure 8:**
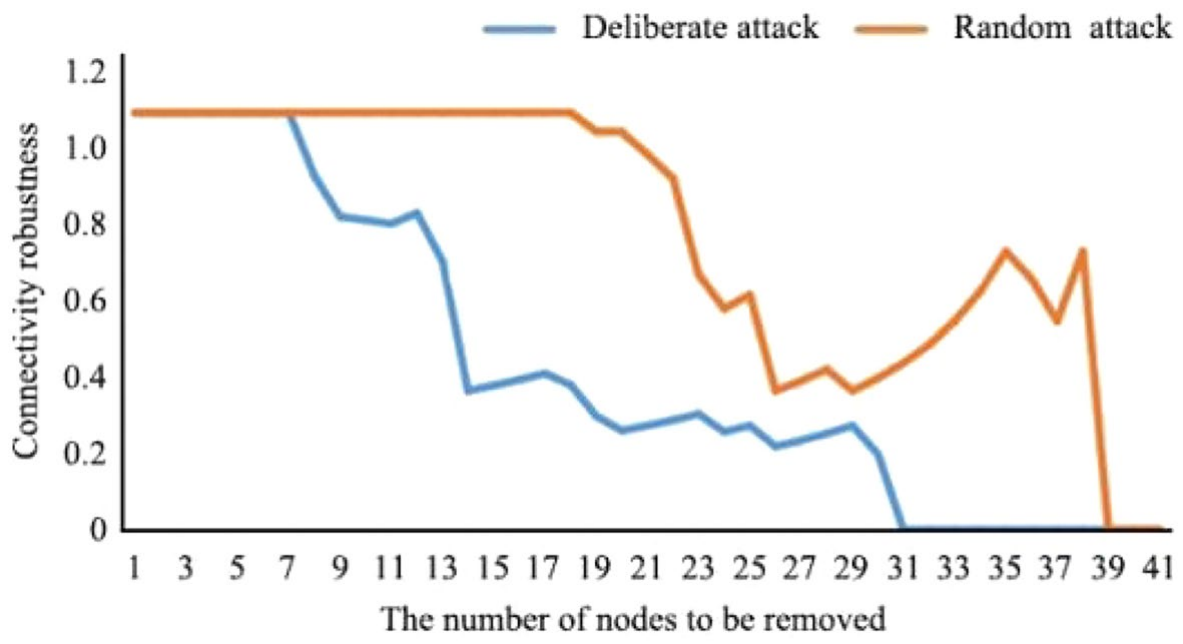
Current ecological spatial network connectivity robustness changes.

**Figure 9:**
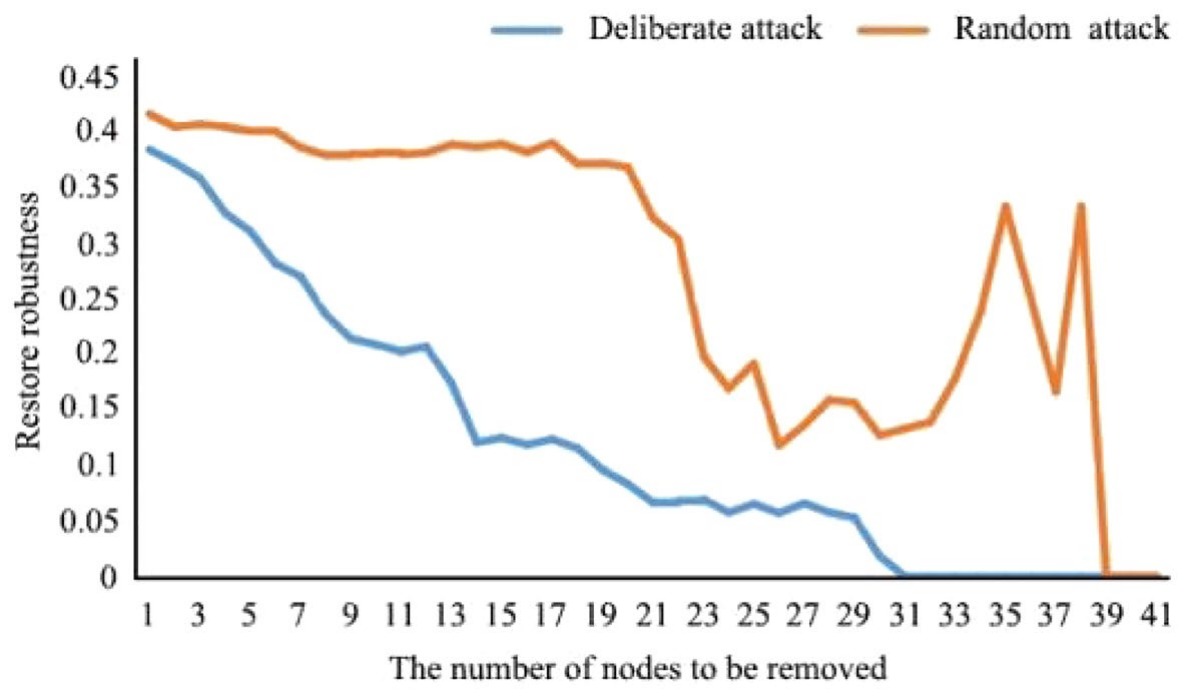
Current ecological spatial network restore robustness changes.

As shown in Figs. 8 and 9, in the scenario with the greatest disturbance, 17.07% of nodes could be deleted without affecting the connectivity robustness of the network. At this time, the network was relatively complete, and the ecological service function was not significantly affected. When 73.17% of the nodes were deleted, the robustness of network connectivity was 0, and the energy flow of the ecological spatial network was basically lost. In the random scenario of natural evolution, when 43.90% of nodes were deleted, the connectivity robustness of the network was not affected, and the network was relatively complete. When 90.27% of the nodes were deleted, the network connectivity robustness was 0, and the ecological spatial network collapsed.

In the future, under the extreme disturbance scenario, when 14.29% of nodes are deleted, the connectivity robustness of the network is not affected, and the network is relatively complete. When 78.57% of the nodes are deleted, the network connectivity robustness is 0, and the energy flow of the ecological spatial network is basically lost. In the future random scenario of natural evolution, when 10.71% of nodes are deleted, the connectivity robustness of the network is not affected, and the network is relatively complete. When 94.64% of nodes are deleted, the connectivity robustness of the network is 0, and the ecological space network collapses. When the network crashes, the number and proportion of disturbed nodes increase compared with that before optimization. Under disturbance, redundancy increases by 5.40%. Under natural evolution, redundancy increases by 4.37%. This result shows that the resilience of the basin to deal with risks is further strengthened, and the numbers of patches and corridors that maintain basic ecological functions and energy flow when the ecological environment is damaged increase.

##### Adaptability

**Figure 10:**
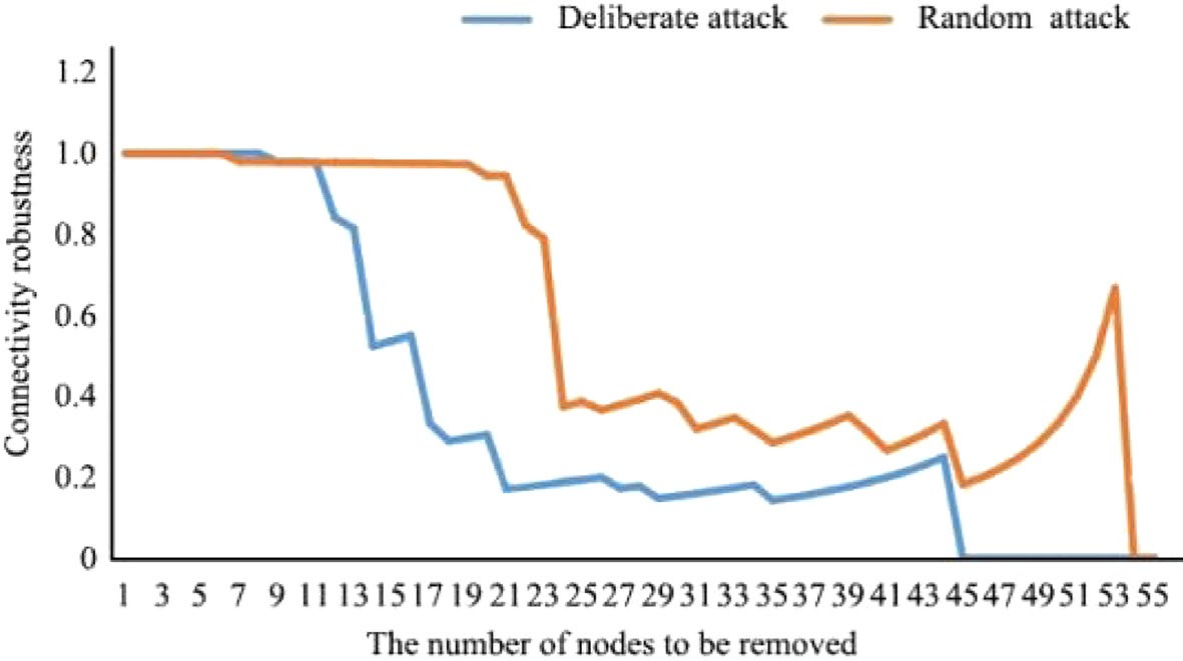
Network connectivity robustness changes in scenario simulation.

**Figure 11:**
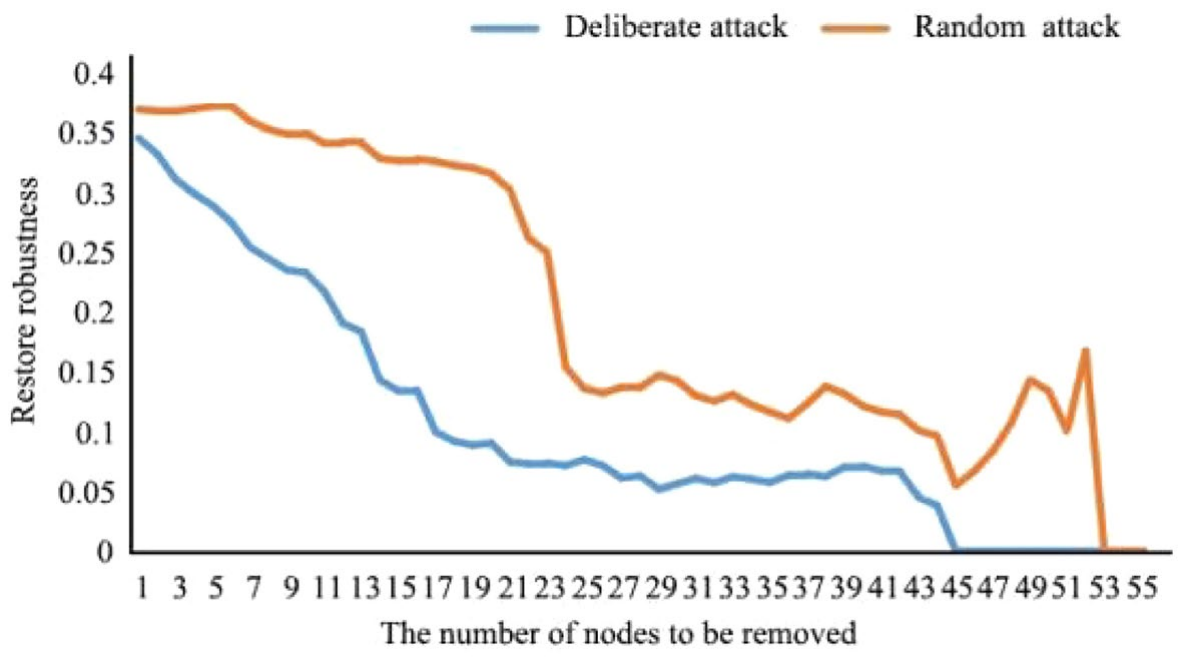
Network restore robustness changes in scenario simulation.

As shown in Figs. 10 and 11, in the extreme scenario of disturbance, when 26.83% of nodes were deleted, the restore robustness of the network decreased more rapidly. At this time, many important ecological patches in the basin were destroyed. When 73.17% of the nodes were deleted, the restore robustness of the network was 0, and the energy flow of the ecological spatial network was basically lost. In the random scenario of natural evolution, when 53.66% of the nodes were deleted, the restore robustness of the network first decreased gently and then decreased sharply. When 92.68% of the nodes were deleted, the restore robustness of the network was 0, and the ecological spatial network collapsed.

In the future, under the extreme disturbance scenario, when 30.36% of nodes were deleted, the restore robustness of the network decreased more rapidly. When 78.57% of the nodes were deleted, the restore robustness was 0, and the energy flow of the ecological spatial network was basically lost. In the random scenario of natural evolution, when 37.50% of the nodes were deleted, the restore robustness of the network first decreased gently and then decreased sharply. When 94.64% of the nodes were deleted, the restore robustness of the network was 0, and the ecological spatial network collapsed. At this time, the number and proportion of disturbed nodes increased compared with those before optimization. Under disturbance, fitness increased by 5.40%. Under natural evolution, fitness increased by 1.96%. Therefore, it can be concluded that when the ecological environment is destroyed, the time needed to maintain efficient material flow in the watershed is extended, and the efficiency of biological flow operation is significantly improved.

### Results of ecological space optimization

#### Ecological space network optimization

**Figure 12:**
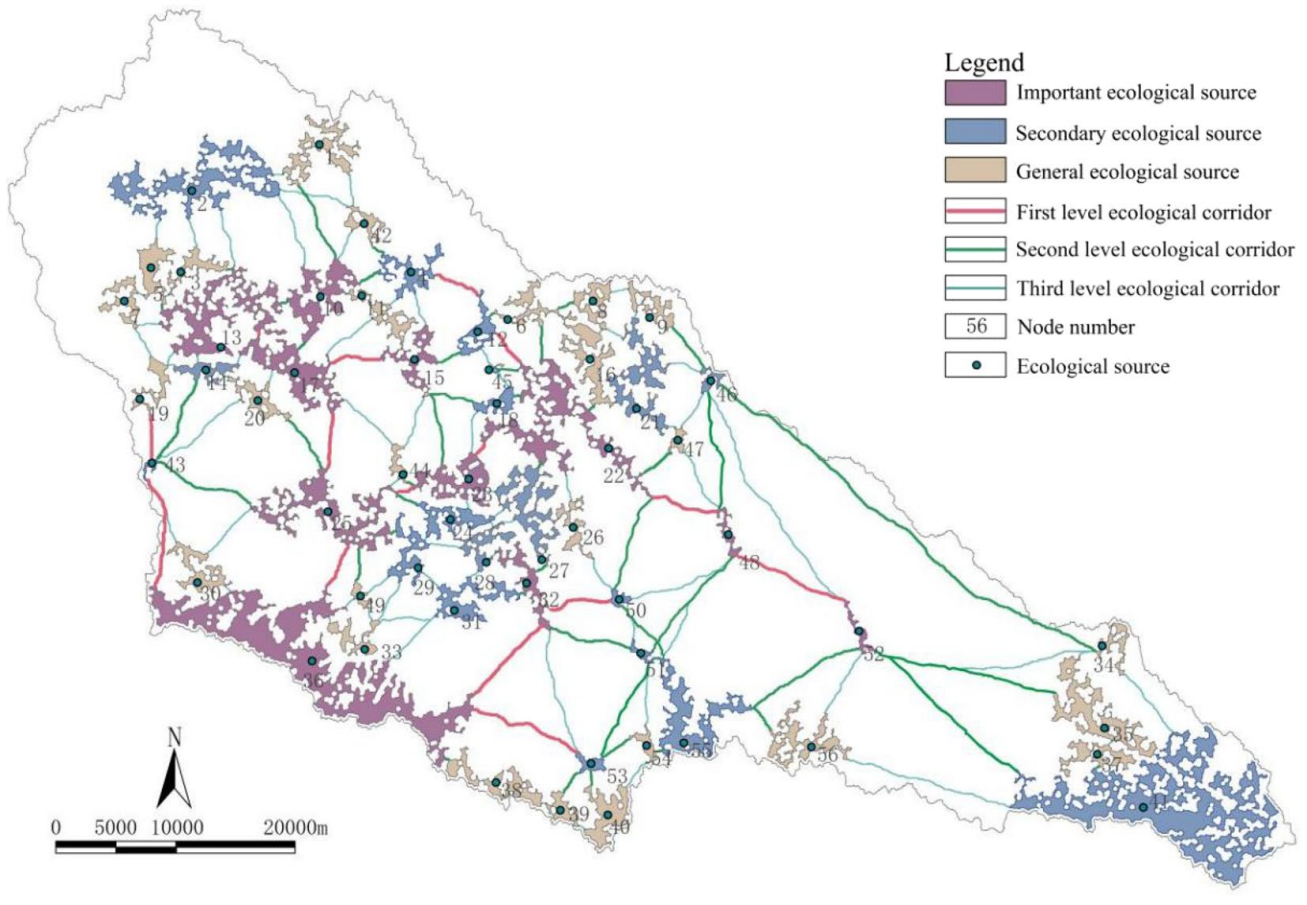
Classification results of the ecological spatial network in the Yanhe River Basin.

As shown in Fig. 12, the importance of ecological source region development was classified into three categories. The primary ecological source regions were located in the middle of the basin and the southern mountainous area, and they focus on the improvement of ecological functions and external penetration. Secondary ecological source regions focus on reducing human disturbance and increasing species diversity. General ecological source regions focus on maintaining existing ecological functions. The specific spatial optimization strategy is shown in Table 3. The importance of ecological corridor maintenance was also divided into three levels. There were 15 first-level ecological corridors connecting primary ecological source regions in series, which played an important role in species richness, migration and dispersal^22^. These corridors should be widened, and the service capacity should be improved. Under the premise of ensuring steady growth in scale, high-quality forest belts with high economic benefits and strong protection effects can be constructed through measures such as higher grafting and rejuvenation. There were 46 s-level ecological corridors covering secondary ecological source regions, which complemented the ecological spatial network. For second-level ecological corridors, their existing service capabilities should be maintained. With the direction of building ecological and functional ecological corridors, spatial optimization should adhere to the suitable forest and grassland areas, promote the connection and upgrading of corridors, and consolidate and expand the achievements of corridor construction. There were 80 third-level ecological corridors, which completed the entire ecological spatial network. There is an urgent need to restore ecological service capabilities to enhance ecological spatial connectivity. Through the combination of natural restoration and artificial promotion, key projects such as comprehensive shelterbelt system construction, the Grain for Green (GFG) program, and natural forest resource protection have been carried out, and they aim to focus on the construction of ecological forest belts, prevent water soil erosion, and promote species migration and energy flow^23^.

**Table 3:** Classification and control table of ecological source regions^24–26^.

Type	Number		Spatial optimization strategy
	Existing nodes	New node	
Primary ecological source region	10, 13, 15, 17, 22, 23, 25, 32, 36	48, 52	Delineate the ecological protection redline in the area where the node is located, consolidate the Grain for Green (GFG) work, strengthen the protection and natural recovery of area, build a forestland buffer zone around the ecological source region, and realize the external penetration of ecological functions
Secondary ecological source region	2, 4, 12, 14, 18, 21, 24, 27, 28, 29, 31, 41	43, 46, 50, 51, 53, 55	Control the growth and encroachment of surrounding farmland, some territorial space development activities unrelated to environmental protection should be restricted, increase vegetation coverage, enrich vegetation types, enhance species diversity, and promote the restoration and connection of regional ecological spatial networks
General ecological source region	1, 3, 5, 6, 7, 8, 9, 11, 16, 19, 20, 26, 30,33,34,35,37,38,39 ,40	42, 44, 45, 47, 49, 54, 56	Protect the ecological source region from encroachment, and carry out ecological restoration of the bare soil areas caused by production and construction

#### Ecological pattern optimization

**Figure 13:**
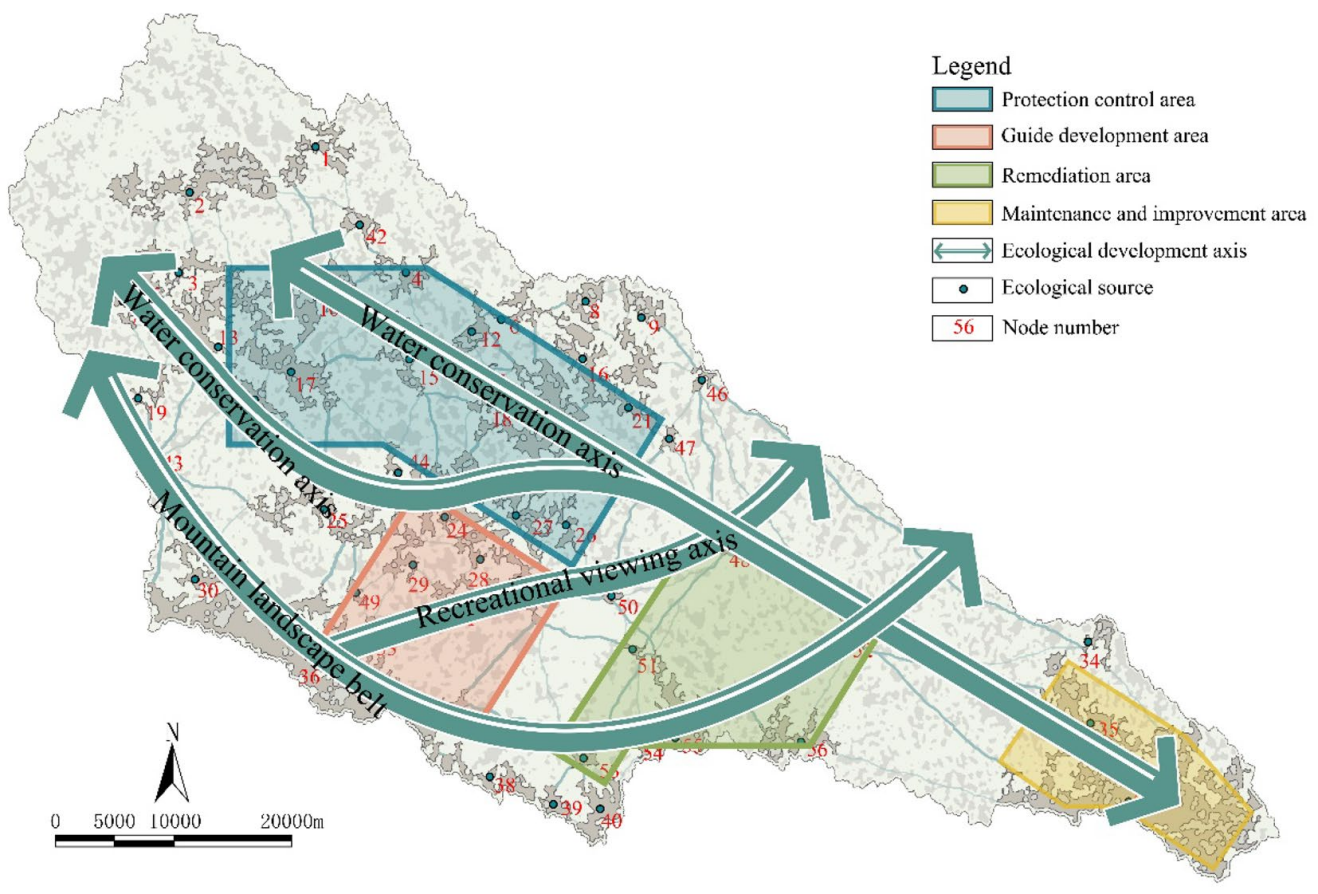
An optimized ecological pattern of the Yanhe River Basin.

The resilience evaluation results of the ecological space in the Yanhe River Basin are shown in Fig. 13. The basin forms the protection control area, the guide development area, the remediation area, and the maintenance and improvement area. For protection control areas, it is necessary to vigorously promote the construction of protected areas, continue closing mountains for afforestation, improve tree diversity and quality, and increase biodiversity. Restrictions on development and construction activities can help reduce water consumption. By building a forestland buffer zone around the water sources, regional water conservation can be achieved. For the guide development area, it is necessary to rely on the Wanhua Mountain Scenic Area and Fodaoping Scenic Area to delineate the scope of protection and development, fully consider the advantages of natural resources, and gradually develop the southwestern mountain forest. For the remediation area, it is necessary to reduce the impact of human activities on the mountain, green the exposed parts of the mountain, and consolidate the achievements of the Grain for Green (GFG) restoration project. For the maintenance and improvement area, it is necessary to improve the resilience of farmland and promote the construction of high-standard farmland. Additionally, a water conservation axis, recreational viewing axis and mountain landscape belt should be created in the basin, and the ecological axis along the Yanhe River should be strengthened to connect more ecological nodes and strengthen the connection between different areas. The water conservation axis connects the Angou Reservoir (41), Wazhuang Reservoir (52), Suntai Reservoir (46), Fangjiahe Reservoir (23), Majiagou Reservoir (15) and Wangyao Reservoir (17) in series. The recreational viewing axis will create a natural landscape belt along the Fodaoping Scenic Area (33) and Wanhua Mountain Scenic Area (31) and penetrate both sides. By adding new nodes 43, 53, 54, and 55, the mountain landscape belt will gradually connect to the ecological node of the mountain on the south side of the basin with the largest primary ecological source region (36).

## Discussion

The ecological nodes of the basin are related to each other, and some key nodes play important ecological functions. For example, the core nodes Wangyao Reservoir (17) and Fangjiahe Reservoir (23) are in the core area of the river basin, with strong ecological service capabilities. Although node 36 is located at the edge of the basin, it still plays an important ecological function. The newly added nodes of ecological forest (48) and Wazhuang Reservoir (52) are small in size, they play an important role in the energy flow and species migration of the entire system, which needs to be protected and controlled during planning. The complex network method can quantify the complex changes in the ecological space, evaluate the ecological functions of different nodes and carry out differentiated control. Therefore, this study conducted scenario simulation and topology simulation on the ecological space of the basin, evaluated ecological nodes and corridors based on 7-type indicators, and proposed an optimization strategy for classification.

Spatial resilience research based on landscape ecology focuses on the spatial attributes and spatial connections of a system^27^. Different from the traditional method based on spatial attribute overlay to study landscape patterns, in this paper, we can identify the nodes that have an important impact on the resilience of ecological space from the perspectives of structure and ecological processes. As far as ecological network structural optimization is concerned, nodes are the crux of network connectivity so that their quantity and quality must be guaranteed^28^. Besides, This study verified the rationality of the indicators. On the basis of enriching the spatial resilience evaluation indicators, this study further divided the indicators from the perspectives of static resilience and dynamic resilience. The key nodes and corridors in the ecological space were found through mathematical models such as node degree, structural hole, and betweenness. For different types of nodes and corridors, differentiated management and control can be carried out separately. Through connectivity robustness and restoration robustness, the changes in the spatial resilience of ecological space under different scenarios were evaluated. It should be noted that the scenario simulation was only for changes at a certain moment in the future. It is necessary to have a long-term under-standing and simulation of the entire revision development process.

The ecological space in the Yanhe River Basin presented the characteristics of water tendency and regional concentration overall. Therefore, spatial optimization must consider some aspects. Water systems, reservoirs and surrounding areas will play greater ecological roles. The surrounding human activities should be reduced, and protected forests should be built. In addition, the construction of landscape belts along both sides of the river should be strengthened^29^. For the middle reaches of the basin, ecological restoration should be strengthened. In contrast, the remediation area identified in this study should prioritize the Grain for Green (GFG) area and vegetation cover on bare land surfaces^2^. For the upstream and estuary areas of the watershed, the existing Grain for Green (GFG) program achievements should be consolidated, and the types and structures of trees should be adjusted to improve species richness^30^.

Based on complex network theory, this paper conducted research on the resilience evaluation and optimization of ecological space in the basin and conducted scenario simulation and testing on the evolution of ecological space in the future. First, the ecological space within the basin was considered as a whole. Through network means, the evolution of the ecological space of the basin was simulated, and the research paradigm of the ecological space was enriched. Second, this study proposed 5 types of static resilience evaluation indicators from the perspective of ecological background and 2 types of dynamic resilience evaluation indicators from the perspective of ecological disturbance, which enriched the evaluation indicators of basin ecological spatial resilience. Finally, an empirical study was carried out taking the basin of the Grain for Green (GFG) program as an example, and the results provide guidance for ecological restoration and ecological pattern optimization in the basin. This method can be used to identify important ecological nodes in the basin, simulate and identify the ecological patches for priority development and protection in the future, and propose protection strategies according to local conditions. With the Yanhe River Basin as an example, it now has 41 ecological source regions and 82 ecological corridors. Based on circuit theory, 15 ecological pinches were identified, and 59 ecological corridors were added. Through ecological pattern optimization based on resilience evaluation, the scopes of the protection control area, the guide development area, the remediation area, and the maintenance and improvement area were delineated, and corresponding control requirements were proposed. Three types of development axes occurred within the water conservation axis, recreational viewing axis and mountain landscape belt. For ecological spatial networks, ecological source regions were divided into primary ecological source regions, secondary ecological source regions, and general ecological source regions. Furthermore, corresponding optimization strategies were proposed. It must be acknowledged that this study has limitations. In the selection of resistance factors, this study failed to fully consider the different impacts of rivers on the migration of terrestrial and aquatic organisms. In addition, there was a lack of consideration of ecological break points created when roads intersect corridors. Moreover, the widening and optimization range of the ecological corridor needs further research.

In this paper, the existing research methods of ecological spatial resilience are expanded. In terms of ecological spatial network optimization, through circuit theory, ecological pinch points can be identified and the ecological spatial network can be optimized. In terms of resilience evaluation, the resilience of the future ecological spatial network is evaluated by using the indicators of complex networks, and the optimization degree of ecological spatial network resilience is obtained by comparing with the current situation. In the future research, further in-depth research can be carried out on source identification and corridor optimization. The method of combining MSPA and index evaluation can be used to make source identification closer to the actual situation. The definition of corridor width and corridor optimization scope can be taken as further research directions for ecological corridor optimization.

## Materials and methods

With the ecological space of the Yanhe River Basin as the research object, the research framework of “network identification-resilience evaluation-spatial optimization” was constructed. MSPA was used to identify the ecological source region, and an ecological spatial network based on the resistance surface and minimum cumulative resistance model (MCR) was constructed. The network was verified by data such as remote sensing and surface characteristics. The static and dynamic resilience of the ecological space was quantitatively evaluated through the complex network structure. Circuit theory was used to identify ecological pinch points, and the structural changes in the complex network were used to simulate the future spatial and temporal changes in ecological space. Finally, the content of spatial optimization was determined.

### Data and preprocessing

#### Research area

**Figure 14:**
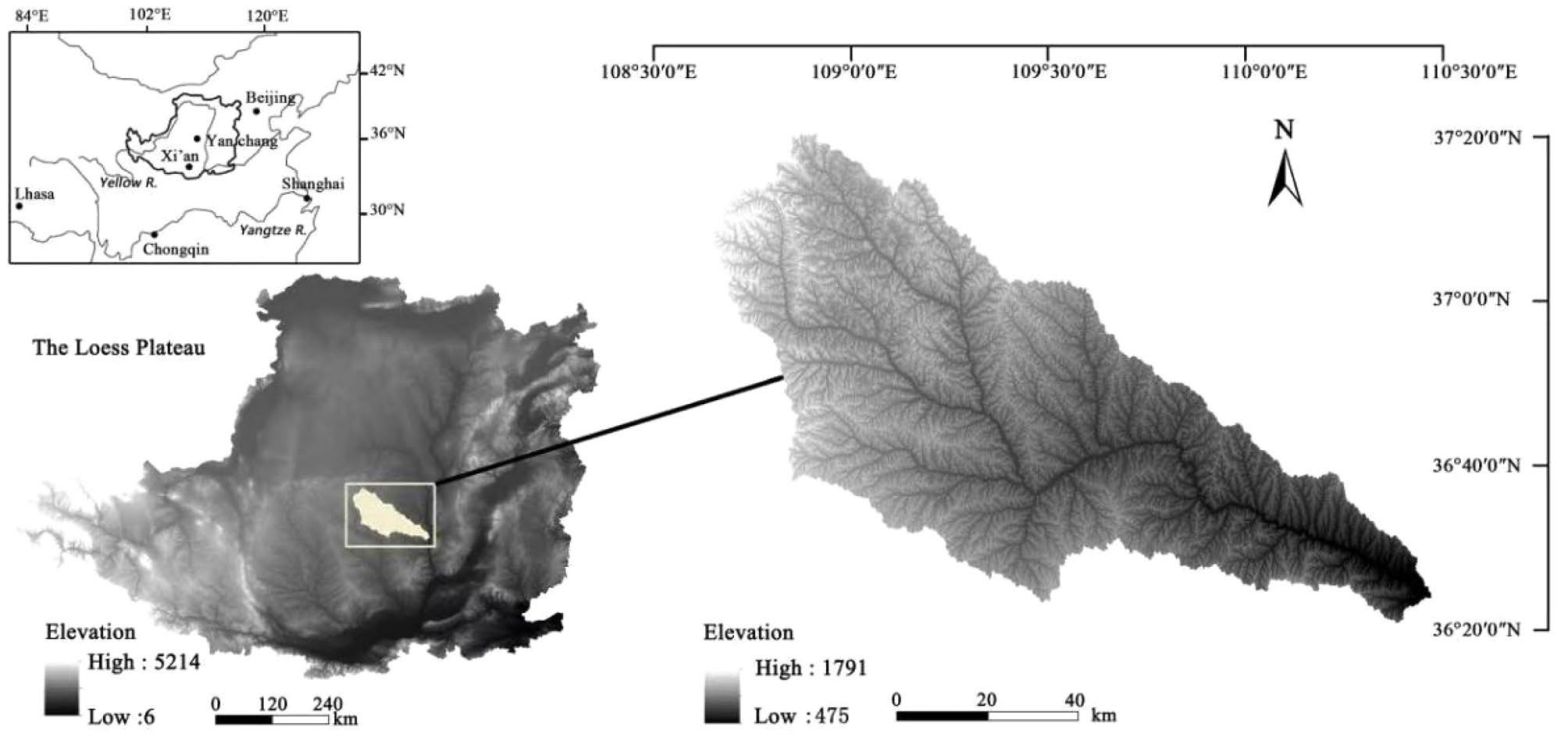
Study area location and elevation.

The Yanhe River Basin has a total area of 7725 square kilometres and an altitude of 1600 m–1823 m, where the Yanhe River runs from northwest to southeast. The region has a continental warm temperate monsoon climate, and the terrain is high in the northwest and low in the southeast, as shown in Fig. 14 (https://www.resdc.cn/).

#### Data sources

The data sources for this study were as follows. The Land-sat8 OLI satellite remote sensing digital product with a 30 m resolution from May 1st to June 31st, 2020, was derived from the geospatial data cloud(https://www.gscloud.cn/). The 30 m resolution digital elevation model data were derived from the geospatial data cloud(https://www.gscloud.cn/). Artificial landscape and scenic reservoir data for the Yanhe River Basin were derived from field studies.

#### Data processing

Land use characteristics: ENVI5.1 software was used for radiometric calibration, atmospheric correction, clipping and supervised classification of remote sensing images. According to the Classification Guidelines for Land and Sea Use in Territorial Spatial Planning and the actual situation of the Yanhe River Basin, land use was divided into forestland, grassland, water area, cultivated land and other land. Among them, ecological space refers to land use types including forestland, grassland, and water area.

Normalized difference vegetation index: ENVI5.1 software was used for radiometric calibration, atmospheric correction and clipping of the remote sensing images(https://envi.geoscene.cn/). However, the normalized difference vegetation index was calculated. Through data error correction, the value range of NDVI is [-1,1], as shown in the formula^31^.

1 $$NDVI = (NIR - R)/(NIR + R)$$

### Identification of the ecological spatial network

#### Ecological source identification

The land use data were reclassified and then imported into Guidos Toolbox software(https://forest.jrc.ec.europa.eu/en/activities/lpa/gtb/) for landscape pattern analysis, in which core areas, bridge areas, edge areas, pores, island patches, branch lines, and round island areas were identified^32^. As an important habitat patch, the larger the core area is, the higher the ecological quality. For the top 50 ecological patches in the core area, Conefor2.6 (http://conefor.org/) was used to calculate the patch importance index (*dPC*), as shown in the formula^33^.

2 $$PC = {{\left( {\sum\limits_{i = 1}^{n} {\sum\limits_{j = 1}^{n} {P_{ij}^{*} \cdot a_{i} \cdot a_{j} } } } \right)} \mathord{\left/ {\vphantom {{\left( {\sum\limits_{i = 1}^{n} {\sum\limits_{j = 1}^{n} {P_{ij}^{*} \cdot a_{i} \cdot a_{j} } } } \right)} {A_{L}^{2} }}} \right. \kern-0pt} {A_{L}^{2} }}$$

3 $$dPC = (PC - PC_{remove} )/PC \times 100\%$$

where *n* is the number of core areas. *a*_*i*_ and *a*_*j*_ are the areas of the habitat patches *i* and *j*. *p*_*ij*_ is the maximum product probability of all possible paths between patches *i* and *j* (including single-step paths). *A*_*L*_ is the total value of the landscape in the study area. *PC* represents the possible continuous interpretation index of a certain landscape, and the value ranges from 0 to 1. The larger the *PC* value is, the higher the connectivity of the landscape plate. *dPC* represents the importance of the patch, and *PC*_*remove*_ represents the possible connectivity index after deleting a certain patch^34^.

Based on the study scale and the actual distance between the patches in the study area, the distance threshold was set to 2500, and the connectivity probability was set to 0.5. Core patches with dPC > 1 were selected as ecological source regions.

#### Extraction of ecological corridor

In this study, the minimum cumulative resistance model (MCR), which quantifies ecological processes in terms of expended cost distance, was used to extract the ecological corridor. During the construction of the resistance surface system, this study selected land use types, distances from water bodies, elevations, slopes, and NDVIs as the index factor systems for resistance surface evaluation (Table 4)^35^. For each resistance factor, a corresponding single-factor resistance surface was constructed. Through the Analytical Hierarchy Process (AHP) method, the weight of each factor was determined^36^. The single-factor resistance surface was then overlaid by the mosaic to a new raster tool to obtain the composite resistance surface of the Yanhe River Basin.

**Table 4:** Ecological resistance factor assignment table.

Evaluation factor	Assignment					Weight
	1	2	3	4	5	
Land use type	Forestland	Grassland	Water area	Cultivated land	Other land	0.51
Altitude	475–800	800–1000	1000–1200	1200–1400	1400–1791	0.14
Slope	< 5°	5°–15°	15°–25	25°–35	> 35°	0.14
Distance from the river	< 100 m	100–200 m	200–500 m	500–1000 m	> 1000 m	0.06
NDVI	0.74–1	0.6–0.74	0.39–0.6	0–0.39	< 0	0.15

All potential ecological corridors in the area were determined by the cost distance and cost back link between each source and the remaining target sources calculated based on the composite resistance surface, as shown in the formula^37^.

4 $$MCR = {\text{f}}_{{{\text{min}}}} \sum\limits_{j = n}^{{{\text{i}} = m}} {D_{ij} \times R_{i} }$$

where *MCR* is the minimum cumulative resistance model. *f* is a function reflecting the proportional relationship between *MCR* and variables *D*_*ij*_ and *R*_*i*_. *D*_*ij*_ indicates the spatial distance between ecological source regions *i* and *j*. *R*_*i*_ indicates the resistance value of patch *i* during species migration.

#### Scenario simulation

Scenario simulation refers to the description of complex and uncertain future possible situations through the simulation of future risk analysis and risk response measures^38^. According to the characteristics and trends of ecological space changes in the Yanhe River Basin in the past 50 years, as well as the specific requirements for territorial spatial planning, the Grain for Green (GFG) project, and ecological restoration projects, the future ecological spatial pattern will rely on natural development, ecological priority, and economic priority to form new connections (Table 5) ^39^. The scenario of natural development refers to the expansion of ecological space caused by ecological processes such as wetland expansion and bare land greening. The ecological priority scenario refers to the possible situation of ecological construction, such as GFG and ecological restoration work. The economic priority scenario refers to the park construction and environmental governance situation developed for economic construction.

**Table 5:** Ecological space scenario simulation table.

Scenario of natural development	Scenario of ecological priority	Scenario of economic priority
Species migration	the Grain for Green (GFG)	Rural park construction
Raw plaque area amplification	Construction of nature reserves	Urban development and construction
Restore naked	Close hillsides to facilitate afforestation	Relocation of rural residents
Vegetation richness	Ecological protection red line division	Urban development boundary division
Slope greening	Construction of constructed wetlands	Riverside ecological landscape construction
Migration corridor protection	Forest park construction	Expressway green belt construction
Natural water storage	Reservoir construction	Rural environmental improvement
Wetland expansion	Add other ecological plaques	Farm construction

The new nodes in the process were determined by the location and size of the ecological pinch points. Based on circuit theory and sports ecology, this study used the Pinchpoint module in the Linkage-mapper tool to identify pinch regions. The position and size of the ecological pinch points were determined by the ecological patch with a certain area and the importance of the patch. Among them, electric charges represent species, conductive surfaces represent resistance surfaces, circuit nodes represent habitats, and pinches represent regions with high probability or irreplaceable passage of species during migration^40^. To better show the position of the pinch point, 1 km was set to the corridor width. Finally, the node and edge information from future simulation results of the watershed ecological space were imported into Pajek (http://mrvar.fdv.uni-lj.si/pajek/) for computational analysis. The final result was painted in Arcgis 10.5 (https://www.esri.com/) and Adobe Photoshop 2021 (https://www.adobe.com/cn/).

### Network resilience evaluation

#### Static resilience evaluation

##### Diversity

Diversity is an indication of the hierarchical nature of a node and the ability to maintain a high level of functionality after some nodes are disconnected. The node degree reflects the influence and importance of the node in the topological network. The larger the degree is, the more important it is in the region, and the stronger the diversity ^20^. Node degree *Ek*_*i*_ is the number of edges connected to node i in the network, e_ij_ represents the edges of the connected node i with node j, and F is the set of all the edges in the network^41^, as shown in the formula:

5 $$E{\text{k}}_{{\text{i}}} = \sum\nolimits_{{{\text{ij}} \in E,{\text{i}} \ne {\text{j}}}} {{\text{e}}_{{{\text{ij}}}} {\text{e}}_{{{\text{ij}}}} \in F}$$

##### Collaboration

Collaboration represents the ability of ecological nodes to coordinate and communicate with different subjects. A structural hole can be understood as a gap between two unconnected nodes that is filled when the two are connected through a third node. Node structural holes reflect the relative competitive advantage of the nodes in the region. Node structural hole *EC*_*i*_ is the degree of dependence of node *e*_*i*_ on other nodes in the network, which is also called the network constraint coefficient^10^. The smaller the constraint coefficient of node I is, the easier the node becomes a node occupying a structural hole, the more diverse the selection of connections, and the stronger the collaboration between nodes^41^, as shown in the following formulas:

6 $$EC_{{{\text{ij}}}} = \left( {P_{{{\text{ij}}}} + \sum {_{{\text{q}}} P_{{{\text{iq}}}} P_{{{\text{qj}}}} } } \right)^{2}$$

7 $$EC{}_{{\text{i}}} = \sum {_{{\text{j}}} EC_{{{\text{ij}}}} }$$

where node *q* is the common adjacent point of node *i* and node *j*. *P*_*ij*_ represents the weight ratio of node* j* in all adjacent points of node *i*.

##### Interdependence

Interdependence refers to the ability of a node, as part of an interconnected and integrated network, to establish functional and physical relationships with other nodes and to gain support therefrom. Clustering coefficients are used to describe the “tightness” of nodes in the network. The higher the clustering coefficients are, the higher the degree of clustering around the nodes and the stronger the interdependence^20,41^, as shown in the following formulas:

8 $$EC{\text{c}}_{{\text{i}}} = 2E_{{\text{i}}} /[Ek_{i} (Ek_{i} - 1)]$$

9 $$ECC_{{\text{i}}} = \frac{1}{{\text{n}}}*\sum\nolimits_{{{\text{i}} \in E}} {EC{\text{c}}_{{\text{i}}} ,0} \le ECC_{{\text{i}}} \le 1$$

where E_i_ is the number of edges that actually exist between the adjacent first-level nodes of node i*.* The clustering coefficient is the first layer clustering coefficient, that is, the absolute degree of the first layer neighbour clustering around node i, ECc_i_ is the node clustering coefficient, and ECC_i_ is the overall network clustering coefficient.

##### Stability

Stability represents the ability of ecological spatial networks to stabilize and operate continuously. If all points in one of the subgraphs in the network are adjacent to at least k other points in the subgraph, such a subgraph is called a “k-core”. The higher the ratio of the k value to the k-core is, the more components that are locally stable in the network, and the higher the resilience of the network as a whole^10^.

##### Connectivity

Connectivity is the ability of nodes in ecological spatial networks to connect through each other. In a complex network, this property is reflected by node betweenness and edge betweenness. Node betweenness and edge betweenness are measurement indicators that characterize the global structural properties of ecological nodes and edges in the topological network, which can reflect the service strength and importance of nodes and edges in the entire network. The larger the value of betweenness is, the more important the corresponding nodes and edges are in the entire network, and the stronger the connectivity. At this point, the node acts as a hub in the region^41^, as shown in the following formula:

10 $$E{\text{b}}_{{{\text{ij}}}} = \sum {_{{{\text{st}} \in E{\text{s}} \ne {\text{t}}}} \frac{{{\text{n}}_{{{\text{st}}}} {\text{(e}}_{{{\text{ij}}}} )}}{{{\text{n}}_{{{\text{st}}}} }}}$$

where *Eb*_*ij*_ is the ratio of the number of shortest paths passing through edge *e*_*ij*_ in all shortest paths between nodes to the total number of shortest paths between all nodes. *n*_*st*_ is the number of shortest paths connecting nodes *e*_*s*_ and *e*_*t*_. *n*_*st*_ (*e*_*ij*_) is the number of shortest paths connecting nodes *e*_*s*_ and *e*_*t*_ and passing through edge *e*_*ij*_.

#### Dynamic resilience evaluation

The stability of ecological space is closely related to natural disasters and human activities. In natural disasters, ecological space is randomly destroyed. When affected by human activities, ecological space is subject to deliberate destruction. The damage to the ecological space caused by the two cannot be quickly recovered in a short period of time. In the ecological spatial network, after the corresponding node is deleted, it cannot be recovered. The dynamic resilience of ecological spatial networks was evaluated by simulating extreme scenarios of deliberate disturbance and random scenarios of natural evolution^42^. Extreme scenarios include felling trees in important patches and constructing recreational areas and public works and building highways in the central patch. The ecological nodes were arranged from high to low according to the node degree and point betweenness, and the structural holes were arranged in order from low to high, which were then removed in turn. Random scenarios included fires, floods, earthquakes and animal disease spreads with random spatial patterns^43^. According to the random data generator, ecological nodes were sorted and removed sequentially.

##### Redundancy

Redundancy indicates the degree of redundancy of the eco-spatial network. When interference occurs, redundant nodes can undertake some functions to ensure the operation of the network. Connection robustness was used to measure when the ecological space network lost some ecological patches, ecological nodes or ecological corridors, the network maintained structural integrity and the ability of the remaining elements to transfer matter and energy to each other. We measured redundancy by the percentage of the number of nodes removed at the complete loss of network function, as shown in the following formula^44^:

11 $$R = \frac{{C_{MAX} }}{{n - n^{\prime } }}$$

where *R* is the connection robustness of the ecological space network. *C*_*MAX*_ is the number of nodes in the largest connected subgraph after the network is disturbed and some nodes are lost. *n* is the number of nodes before the network is disturbed. *n’* is the number of nodes lost after the network is disturbed.

##### Adaptability

Adaptability represents the ability of the network to adapt and return to a certain operating state after interference. Restore robustness is used to describe the ability to maintain the efficient operation of biological flow after the ecological spatial network is disturbed, which is usually measured by the global efficiency of the topology network. The higher the global efficiency is, the higher the efficiency of the ecological spatial network to ensure the operation of the biological flow after being distrurbed. We measured adaptability by the percentage of the number of nodes removed when the biological flow completely lost its ability to run efficiently, as shown in the following formula^45^:

12 $$E = \frac{1}{{{\text{n}}{\text{n - }}1}}\sum\limits_{i \ne j \in G} {\frac{1}{{d_{ij} }}}$$

where E is the restore robustness. n is the number of all nodes in the network. G is the set of network nodes. Node i and node j are any two points in network G*.* d_ij_ is the shortest path length from node i to node j.

**Figure 15:**
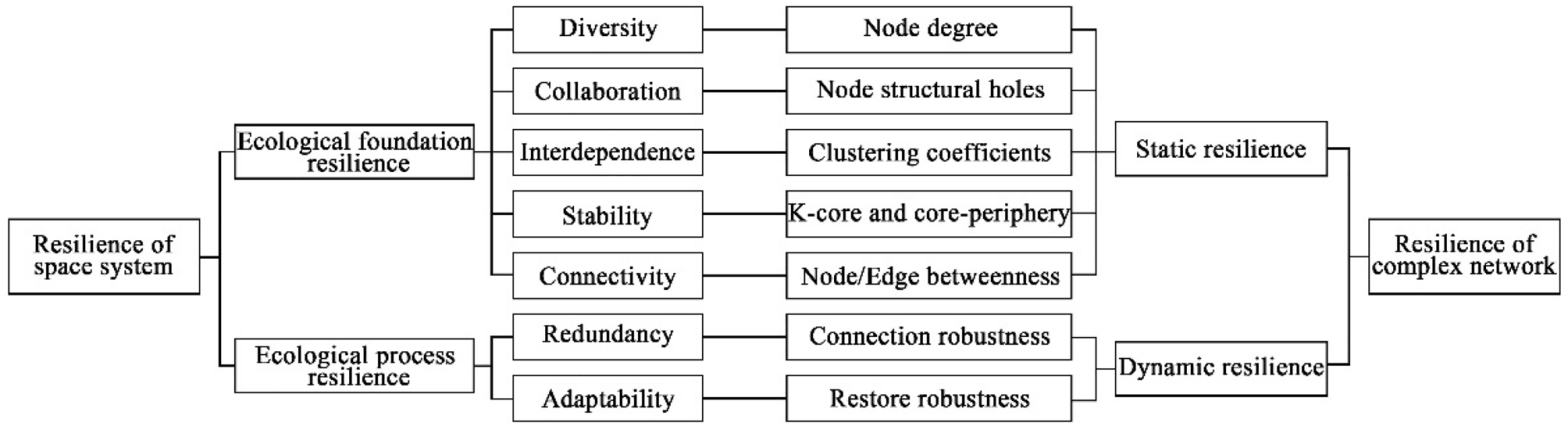
The relationship between spatial resilience and complex network resilience.

The correspondence of spatial resilience to network resilience is shown^46–50^ in Fig. 15.

### Optimization of ecological space

#### Ecological space network optimization

According to the results of the resilience evaluation and the evolution trend of scenario simulation, the ecological spatial network of the basin was optimized. The first step was to classify and control the ecological source regions of the basin and propose control strategies. After calculating the weighted average of the evaluation results and sorting them in descending order, the top 20% were selected as primary ecological source regions, 20% ~ 40% as secondary ecological source regions, and the rest as general ecological source regions. The next step was to carry out hierarchical control of the ecological corridor and provide a reference for ecological pattern optimization. After sorting the edge betweenness in descending order, the top 10% were selected as first-level ecological corridors, 10% ~ 40% as second-level ecological corridors, and the rest as third-level ecological corridors.

#### Ecological pattern optimization

The ecological pattern optimization of the Yanhe River Basin needs to comprehensively consider the Grain for Green (GFG) policy, territorial spatial planning, evolution of ecological space, and hierarchical control strategies of ecological spatial networks. The first-level ecological corridors where the series reservoirs are located were defined as the water conservation axis. The first-level ecological corridors where the hilly forestland is located were designated as the mountain landscape belt. The first-level ecological corridors that connect the source of tourist attractions were designated as the recreational viewing axis. The concentrated area of the primary ecological source regions where the upstream reservoir is located was divided into protection control areas. The concentrated area of the primary ecological source regions where the scenic spot is located was divided into guide development areas. The concentration area of newly added important and secondary ecological source regions was divided into remediation areas. The large concentration areas of the downstream secondary and general ecological source regions were divided into maintenance and improvement areas. Combined with the simulation results of the scenario analysis, the evolution trend of the ecological space structure in the Yanhe River Basin was identified.

## Data Availability

All data supporting the findings of this study are available within the article.
